# P03-006 - Pamidronate rapidly decreases CRP and TNFΑ in CNO

**DOI:** 10.1186/1546-0096-11-S1-A201

**Published:** 2013-11-08

**Authors:** MM Kostik, LI Vasyakina, VM Ponomarenko, IA Chikova, AV Minkin, OV Kalashnikova, IV Shvedovchenko, VG Chasnyk

**Affiliations:** 1 Hospital Pediatry, Saint-Petersburg State Pediatric Medical University, Saint-Petersburg, Russian Federation; 2 Department of Immunology, Federal Institute of Public Health, The Nikiforov’s Russian Center of Emergency and Radiation Medicine, Saint-Petersburg, Russian Federation; 3 St. Petersburg Scientific and Practical Center of Medical and Social Expertise, Prosthetics and Rehabilitation n.a. G.A. Albrecht , Saint-Petersburg, Russian Federation

## Introduction

Pediatric chronic nonbacterial osteomyelitis (CNO) is a sterile inflammatory bone disorder of presumed autoimmune or autoinflammatory etiology. Sometimes CNO associated with other rheumatologic conditions, such spondyloarthritis, sacroileitis, IBD, pyoderma gangrenosum and psoriasis as well as a part of distinct autoinflammatory disease (AID), such as DIRA syndrome.

## Case report

14 year old girl was admitted to our department with pain, low and moderate grade fever, delay to thrive and history of osteomyelitis. Her family history was unremarkable. The onset of disease was in the age of 10 months with small bone lesion in distal epiphysis of femur and intensive irritability. After 1 month of immobilization she had an intensive bone overgrowth with periostal reaction and deformity in distal epiphysis. The bone biopsy confirmed non-specific osteomyelitis, malignancy and infection was excluded. Antibiotic treatment was ineffective. Her disease had wide-spread course involving the whole femur from distal to proximal part. The main features included bone lesions, bone overgrowth, intensive periostal reaction and sclerosis. The disease had persistant course without remission episodes, accompanied with pain, irritability, fever and lead to elongation of femur (+8 cm). She had failure to thrive since the age of 6 years. Currently she looks like as 8 year old girl in her 14 years. Intellectual development is normal. No signs of any other diseases, particularly recurrent infections and involvement of other bones. A Tc99m bone scan revealed increased uptake only in the whole femur (+400%). Laboratorial features were specific for AID: persisted microcytic anemia (Hb-8.0 gr/dl), ESR>110 mm\h (n.v.<15), CRP >150 mg/l (n.v.<5), sideropenia. Immunological assessment was detected increased Ig A (5.2 gr\l), Ig G (26.2 gr\l) and decreased zymozan-induced chemoluminescence (11 Units, lower limit -160). Chronic granulomatose disease (CGD) was confirmed without any known foci of serious infection during her life. Also high levels of IL1β, IL-6 and TNFα were detected. NSAIDs appeared short temporary effect.

In our clinic we started to treat her with pamidronate 1,5 mg/kg on 1 cycle, with monthly repeated courses. After first cycle the CRP was decreased from 150 to 19 mg/l, ESR from 58 to 12 mm\h, TNFα from 169 to 19 pg\ml. Hb increased up to 10.2 gr/dl. No pain, irritability and fever after initiating pamidronate therapy.

## Discussion

We describe a case of early presented chronic nonbacterial osteomyelytis affected of only one bone. Early onset (first year of life), permanent progredient course, systemic features (fever, failure to thrive), typical laboratorial changes (microcytic anemia, very high ESR and CRP), increased levels of IL1β, IL-6 and TNFα are characteristic for AID. The described case can be new form of an AID with clinical features resembling CRMO or DIRA diseases. Biologics can be considered to be a promising way of further treatment as it has been reported to be successful already for small number of cases.

## Competing interests

None Declared.

